# Pityriasis versicolor chez des nourrissons: aspect clinique inhabituel et rôle de la dépigmentation cosmétique par des corticoïdes chez la mère

**DOI:** 10.11604/pamj.2017.26.31.11504

**Published:** 2017-01-23

**Authors:** Pauline Dioussé, Fatimata Ly, Mariama Bammo, Sarah Lizia, Thierno Abdoul Aziz Diallo, Haby Dione, Fatou Sarr, Ramatoulaye Diagne Gueye, Amadou Mactar Gueye, Mame Thierno Dieng, Bernard Marcel Diop, Mamadou Mourtalla Ka

**Affiliations:** 1 UFR des Sciences de la Santé, Université de Thiès, Sénégal; 2 Dermatologie, Université Cheikh Anta Diop, Dakar, Sénégal; 3 Dermatologie, Hôpital Régional de Thiès, Sénégal

**Keywords:** Pityriasis versicolor, dermocorticoïdes, nourrisson, membres inferieurs, Pityriasis versicolor, dermocorticoids, infant, lower limbs

## Abstract

Nous rapportons deux observations de *Pityriasis versicolor* (PV) chez des nourrissons âgés de 12 etde 18 mois. Ces derniers étaient amenés en consultation pour des macules hypochromiques et achromiques, rondes, localisées aux membres et sur le visage. On retrouvait une notion de dépigmentation volontaire à visée cosmétique à base de corticoïdes et d’hydroquinone chez leur mère depuis en moyenne 5 ans. Le scotch test effectué chez un des nourrissons et sa mère montrait de courts filaments et des grappes de spores. Le traitement était à base de kétoconazole. Après 8 semaines, l’évolution était favorable chez tous les patients avec cependant une persistance de quelques macules hypochomiques. Les particularités de nos observations sont d’une part la topographie aux membres inférieurs et d’autre part le caractère familial du PV dont la survenue est favorisée par l’usage de produits dépigmentant à base de corticoïdes. Ces derniers favorisent le caractère atrophique et achromique des lésions. En effet, les formes achromiques des membres inférieurs étaient décrites chez les adultes s’adonnant à la dépigmentation artificielle.

## Abstract (en)

We report two cases of Pityriasis versicolor (PV) in infants aged 12 and 18 months. The latter were brought to medical attention because of hypochromic and achromic, round macules involving the limbs and the face. Physical examination of their mothers showed voluntary depigmentation for cosmetic purposes due to the use of corticosteroids and hydroquinone, on average, over a 5-year period. The scotch tape test performed in one of the infants and his mother showed short filaments and clusters of spores. Treatment was based on ketoconazole. After 8 weeks, all patients reported favorable outcomes despite the persistence of some hypochomic macules. The peculiarities of this study are, on the one hand the topography of the lower limbs and on the other hand a positive family history of PV whose occurrence is favored by the use of depigmenting agents based on corticosteroids. Corticosteroids favor the atrophic and achromic feature of the lesions. Indeed, achromic lesions on the lower limbs were described in adults undergoing artificial depigmentation.

## Introduction

Le pityriasis versicolor est une mycose superficielle causée par les levures lipophiles du genre Malassezia [1]. Il atteint préférentiellement les adultes, plusieurs facteurs sont suspectés dans sa survenue parmi lesquels des facteurs environnementaux, génétiques et l’immunodépression. Il est rarement rapporté chez les nourrissons chez qui aucun facteurfavorisant n’a été clairement identifié [2]. Nous rapportons deux observations de PV chez desnourrissons dont la mère pratiquait une dépigmentation à visée cosmétique à base de dermocorticoïdes.

## Patient et observation

**Figure 1:**
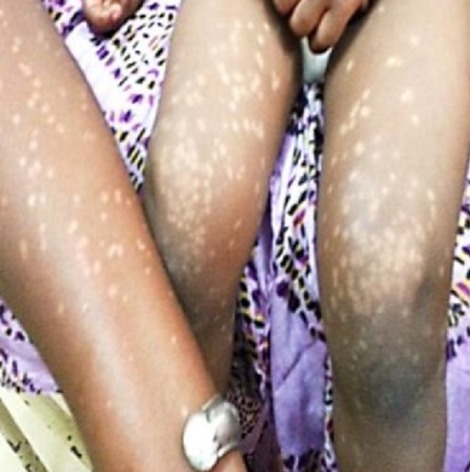
Macules achromiques, rondes, de *Pityriasis versicolor* localisées aux membres inférieurs chez l’enfant et à l’avant-bras chez la mère (observation 1)

**Figure 2:**
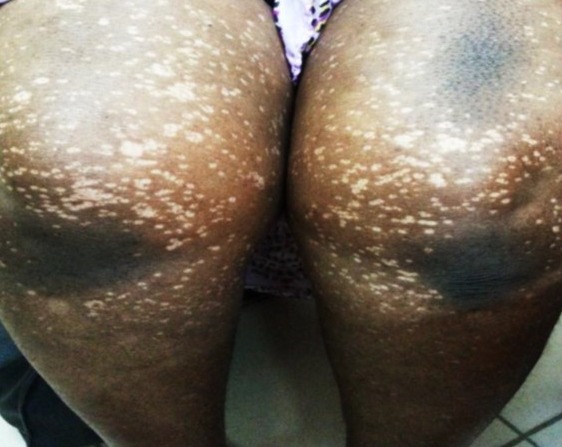
Macules achromiques de *Pityriasis versicolor* des membres inférieurs chez la mère sur terrain de dépigmentation artificielle (observation 1)

**Figure 3:**
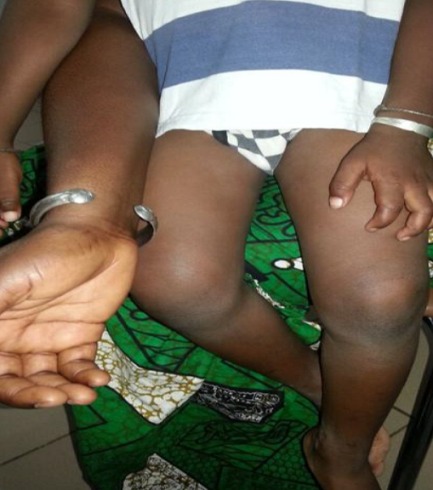
Disparition complète des lésions après traitement (observation 1)

**Observation n°1:** un nourrisson de 18 mois était reçu pour des lésions achromiques des membres évoluant depuis 4 mois. L’examen notait des macules achromiques, rondes, de taille différente, localisées aux membres supérieurs mais prédominant aux membres inférieurs (Figure 1). Il n’existait pas de prurit, aucun terrain particulier n’était retrouvé; les vaccinations étaient à jour et l’allaitement était de type maternel. Des lésions similaires étaient retrouvées chez la mère au niveau du tronc, des membres supérieurs mais prédominaient aux membres inférieurs (Figure 2). La mère pratiquait de la dépigmentation artificielle depuis 5 ans avec des produits à base d’hydroquinone et de propionate de clobétasol. Un scotch test effectué chez l’enfant et la mère, montrait de courts filaments et des grappes de spores. Le traitement était à base de kétoconazole en crème une fois par jour et en gel moussant en application locale (2 fois par semaine). Après 8 semaines, les macules avaient complétement régressé laissant en place une hypochromie (Figure 3).

**Figure 4:**
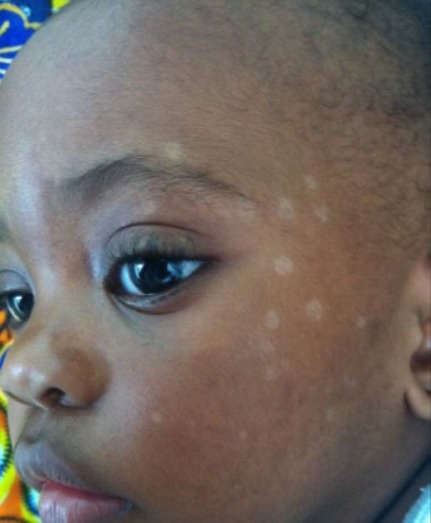
Lésions hypopigmentées et squameuses du visage de l’enfant (observation 2)

**Figure 5:**
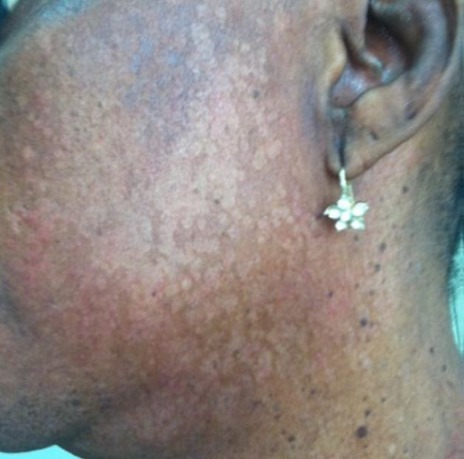
Macules hypopigmentées, squameuses du visage de la mère (observation 2)

**Observation n°2:** un nourrisson de 12 mois était amené en consultation pour des macules prurigineuses dont le début remontait à 3 mois après la naissance (Figure 4). L’examen retrouvait des macules hypochromiques voire achromiques siégeant au visage. Des lésions similaires étaient retrouvées chez la mère âgée de 43ans et qui évoluaient depuis 05 ans (Figure 5). Les antécédents étaient sans particularités. L’enquête cosmétologique chez la mère révélait une pratique de dépigmentation cosmétique depuis 10 ans avec des produits contenant des corticoïdes (Propionate de Clobétasol) et de l’hydroquinone. L’examen retrouvait des lésions similaires à celles retrouvées chez l’enfant au niveau du tronc, cou et visage. Le diagnostic de PV familial était retenu et un traitement à base de kétoconazole prescrit; un arrêt des produits dépigmentant était conseillé. L’évolution était favorable sur le prurit au bout de tandis que les macules hypochromiques régressaient beaucoup plus lentement.

## Discussion

Nous avons rapporté deux observations de PV survenant chezdes nourrissons dont les mères pratiquaient une dépigmentation cosmétique avec des produits dépigmentant à base de corticoïdes. Le caractère achromique des lésions ainsi que la topographie aux membres inférieurs constituent des particularités de nos observations. Habituellement le PV est le plus souvent diagnostiqué chez les adolescents, lorsque l´activité de la glande sébacée augmente [1, 3]. Dans la littérature, la moyenne d’âge de survenue du PV est de 11 ans [3]. Dans certaines séries, la localisation préférentielle de la face, la prédominance du type hypochromique sont des caractéristiques du PV sur peau pigmentée [1, 3, 4]. La topographie aux membres inférieurs a été rapportée chez les adultes s’adonnant à la dépigmentation artificielle [5] mais elle est exceptionnelle chez l’enfant. Il en est de même de la forme achromique qui est plutôt associée à l’usage de dermocorticoïdes [6]. Parfois, une application de dermocorticoïdes a été rapportée chez des nourrissons dont la mère pratiquait une dépigmentation volontaire. Cependant, les contacts étroits et prolongés entre la mère et son enfant ont pu favoriser l’exposition des nourrissons aux dermocorticoïdes. Nous pouvons également évoquer une forme familiale de PV au cours de laquelle des facteurs génétiques sont incriminés. Le traitement topique est indiqué en première intention, en cas d’échec, l´itraconazole ou le fluconazole peuvent être prescrits [1, 2]. Une guérison est obtenue dans un délai de 03 à 04 semaines comme ce fut le cas chez nos patients. Toutefois, les troubles dyschromiques peuvent persister pendant plusieurs semaines.

## Conclusion

Nos observations de PV sont originales par l’âge de survenue chez des nourrissons, la topographie aux membres inférieurs et le contexte de dépigmentation artificielle par le propionate de clobétasol chez la mère.
